# High Resolution Imaging of Vascular Function in Zebrafish

**DOI:** 10.1371/journal.pone.0044018

**Published:** 2012-08-30

**Authors:** Simon C. Watkins, Salony Maniar, Mackenzie Mosher, Beth L. Roman, Michael Tsang, Claudette M. St Croix

**Affiliations:** 1 Department of Cell Biology, The University of Pittsburgh, Pittsburgh, Pennsylvania, United States of America; 2 Department of Environmental and Occupational Health, The University of Pittsburgh, Pittsburgh, Pennsylvania, United States of America; 3 Department of Biological Sciences, The University of Pittsburgh, Pittsburgh, Pennsylvania, United States of America; 4 Department of Developmental Biology, The University of Pittsburgh, Pittsburgh, Pennsylvania, United States of America; Katholieke Universiteit Leuven, Belgium

## Abstract

**Rationale:**

The role of the endothelium in the pathogenesis of cardiovascular disease is an emerging field of study, necessitating the development of appropriate model systems and methodologies to investigate the multifaceted nature of endothelial dysfunction including disturbed barrier function and impaired vascular reactivity.

**Objective:**

We aimed to develop and test an optimized high-speed imaging platform to obtain quantitative real-time measures of blood flow, vessel diameter and endothelial barrier function in order to assess vascular function in live vertebrate models.

**Methods and Results:**

We used a combination of cutting-edge optical imaging techniques, including high-speed, camera-based imaging (up to 1000 frames/second), and 3D confocal methods to collect real time metrics of vascular performance and assess the dynamic response to the thromboxane A_2_ (TXA_2_) analogue, U-46619 (1 µM), in transgenic zebrafish larvae. Data obtained in 3 and 5 day post-fertilization larvae show that these methods are capable of imaging blood flow in a large (1 mm) segment of the vessel of interest over many cardiac cycles, with sufficient speed and sensitivity such that the trajectories of individual erythrocytes can be resolved in real time. Further, we are able to map changes in the three dimensional sizes of vessels and assess barrier function by visualizing the continuity of the endothelial layer combined with measurements of extravasation of fluorescent microspheres.

**Conclusions:**

We propose that this system-based microscopic approach can be used to combine measures of physiologic function with molecular behavior in zebrafish models of human vascular disease.

## Introduction

Our goal in these studies was to develop and test a system-based, high speed microscopic approach that can be used to combine measures of physiologic function with molecular behavior in zebrafish *(Danio rerio)* models of human vascular disease. To this end, we focused on the application of intravital imaging of vascular dynamics in 3–5 days post fertilization (dpf) embryos to investigate the multifaceted nature of endothelial dysfunction [1]. The endothelium plays a well recognized role in modulating vascular resistance and blood flow distribution via production of vasoactive mediators (i.e. NO, endothelin-1); and acts as a semi-permeable barrier between the vascular lumen and surrounding tissue [2]. Endothelial dysfunction results in deleterious consequences to the underlying tissue [2], [3] and, as such, has been associated with a host of vasculopathologies, including atherosclerosis, hypertension, coronary disease, diabetes, heart and renal failure [4]. While optical microscopy has afforded tremendous insight into endothelial function at the molecular and cellular level, visualization of cardiovascular physiology in living vertebrates is a particular challenge as the relevant cells and tissues are relatively inaccessible to microscope based imaging tools. The optical transparency (when grown in 1-phenyl-2-thiourea (*PTU*)) and size of the zebrafish embryo are ideally suited to modern microscopy such that complex biologic processes can be visualized *in toto* and *in vivo*. Importantly, the vascular is anatomically and functionally comparable to other vertebrate species [5]. As such, transgenic zebrafish expressing fluorescent proteins in endothelial lineages, concurrent with vascular specific mutations, have been instrumental in unraveling the complex molecular processes governing vascular development [6], [7]. We posited that the zebrafish embryo is an appropriate vertebrate model to study vascular function as humoral factors and pharmacological agents, including epinephrine [8], [9], nitric oxide (NO) [10] and agonists of the cyclooxygenase/thromboxane pathway [11] have been shown to elicit equivalent effects on cardiac contractility, and/or blood flow in developing zebrafish as seen in humans.

**Figure 1:**
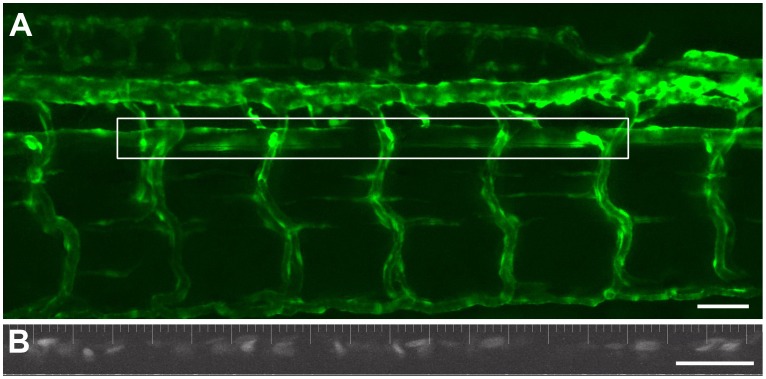
Intravital imaging of vascular dynamics in a 5 dpf zebrafish larva. An extended depth of focus projection (dorsal upwards, anterior left) of the vasculature of a *Tg(kdrl:GFP)^la116^;Tg(gata1:dsRed)^sd2^ embryo with the fluorescence defining the endothelium (* ***A*** *)*. The outlined box shows the area of the aorta used for the time-based imaging of blood flow velocity and vessel diameter. The lower panel (**B**) shows a representative image from the same fish of the DsRed labeled erythrocytes within the dorsal aorta. This image was collected with a 2 ms exposure. With these settings it was readily possible to visualize the red blood cells flowing through the dorsal aorta over time. (Scale Bar  = 100 microns).

Accurate real-time measurement of vascular resistance (vessel diameter) and regional blood flow demands high speed detection methods such that individual erythrocytes can be tracked and flow velocity calculated over a defined segment of vessel. Until very recently, conventional camera technologies were not able to collect images at sufficient speed or resolution to make dynamic measures of flow velocity in living organisms, including zebrafish. High speed, charge coupled devices (CCD) have low pixel counts (typically 128×128 pixels) which compromises resolution. Conventional CCD and electron multiplying CCDs (EMCCD) have higher pixel counts (between 512×512 and 1300×1000), but have total pixel read speeds of 10–20 mHz (10–40 frames/second, fps). Strategies have been developed to circumvent these limitations and obtain gross estimates of blood flow, including time-lapse imaging of endogenous (erythrocytes, platelets) or tracer particles to obtain displacement information between consecutive frames of data [12], [13], [14]. These tracking methods have become increasingly sophisticated with the adoption of digital particle image velocimetry (DPIV) [15] to provide quantitative assessments of flow dynamics [12], [16]. As with traditional erythrocyte tracking methodologies, the utility of DPIV is limited by the temporal resolution of the cameras used and the ability of the system to resolve individual particles as discrete objects within the flow field [17]. Furthermore, these methodologies are mathematically complex and cannot be used for long-term image data collection. Since the beginning of 2011, multiple scientific complementary metal oxide semiconductor (sCMOS) detectors have become commercially available. These cameras have two to four times as many pixels as conventional CCD detectors. More importantly, each column of pixels is read independently, reducing read noise and allowing far higher read rates than contemporary CCD cameras. For these studies, we incorporated the CMOS camera in an optimized microscope platform that was configured to exploit the speed capabilities of the detector. Our objective in these studies was to go beyond the speed/sensitivity limits of existing imaging technologies and collect useful, quantitative fluorescent images at millisecond frame rates and generate real-time, image-based analysis of vascular dynamics within a living vertebrate.

**Figure 2:**
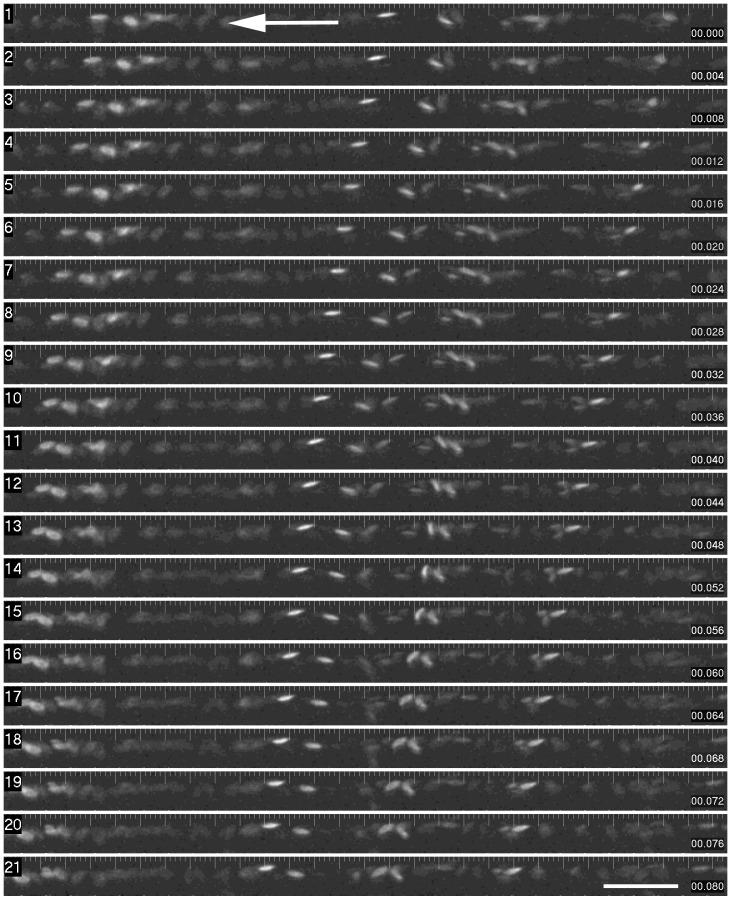
Time sequence of red blood cell flow through a segment of dorsal aorta. This montage shows individual images from a time sequence (collected every 2 ms, with a 2 ms exposure). The images shown are semi-sequential (4 ms intervals). Erythrocytes are readily visible, and the tracking of the cells from right to left can be readily seen. (Scale Bar  = 100 microns).

## Methods

### Zebrafish Lines and Maintenance

Zebrafish (*Danio rerio*) were maintained according to standard protocols [18]. Measurements were made in 3 and 5 day post-fertilization (dpf) embryos generated from a *chrna1^b107/+^;Tg(kdrl:GFP)^la116^;Tg(gata1.dsRed)^sd2^* incross. Transgenic lines *Tg(kdrl:GFP)^la116^* and *Tg(gata1:dsRed)^sd2^* have been previously described [19], [20], [21].The *chrna1* mutants lack functional nicotinic muscle acetylcholine receptors (AChRs) [22]. The mutation acts autonomously in muscle cells and mutant embryos are paralyzed but otherwise develop normally. Only immobile embryos homozygous for the *chrna1^b107^* mutation were imaged, thereby circumventing the need for anesthesia or agar embedment. Embryo medium was supplemented with 0.003% phenylthiourea (PTU) (Sigma, St Louis, MO, USA) at 24 hours post-fertilization (hpf) to prevent melanin formation [23].

**Figure 3:**
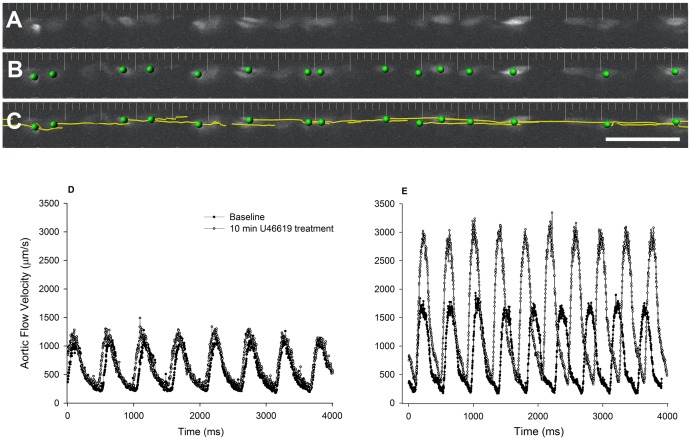
Quantitation of aortic blood flow velocity in zebrafish embryos using intravital imaging. Image processing was performed using Imaris software (Bitplane, Saint Paul, MN). Red blood cells shown in a representative single time point (**A**) were segmented by size, shape, and fluorescence intensity (**B**) and tracked using an auto-regressive tracking algorithm (**C**). The frequency of image collection was set such that erythrocyte displacement is less than one cell diameter/frame and therefore individual cells can be tracked with confidence. Only cells that could be tracked for >250 µm were included in the analysis. Quantitation revealed pulsatility of blood flow in both the 3 (**D**) and 5 (**E**) dpf embryos, and confirmed our ability to measure flow velocities greater than 2 mm/s in response to the thromboxane analogue U46619 (example shown in 5 dpf embryos, panel **E,** open circles).

### Real-time Visualization and Quantification of Blood Flow Velocity

Embryos were placed in a coverslip glass bottomed (#1.5) petri dish (MatTek Corp., Ashland, MA) and mounted in a humidified chamber (Tokai Hit Co., Shizuoka-ken, Japan) atop the motorized stage of a Nikon TiE inverted fluorescent microscope equipped with a 10X, 0.5NA plan apochromat lens (Nikon Inc., Melville, NY), a fluorescent illuminator (89 North, Burlington, VT), DsRed and EGFP filter cubes (Chroma Technology Corp., Bellows Falls, VT) and a Hamamatsu Flash 2.8 CMOS camera (Hamamatsu Corporation). The sensor in this camera has 3.63 micron pixels in a 1920 by 1440 array; the full chip readout is 45 frames/second, each pixel row (1920 pixels) is readout 15 microseconds such that a block of pixel rows consisting of 133 rows can be read at 500 frames/second. The use of a relatively high numeric aperture (NA), low magnification objective allowed large areas to be imaged with a high photon recovery, and a relatively large depth of field. The EGFP labeled vasculature (**Fig. 1A**) was imaged live on the computer monitor. In order to maximize the readout speed of the CMOS camera, the dorsal aorta was aligned with the “column” pixels of the camera. Image sequences were collected using NIS Elements software (Nikon Inc.) using a subarray of pixels (1920×90 pixels) and a 2 ms exposure. Collection speeds of 486 frames per second (fps, close to the theoretical maximum of 500 fps at this exposure time) were achieved by streaming to memory, making it is possible to collect up to 10 seconds worth of data (4860 frames). Given the repetitive nature of the measured events (blood flow velocity) image sequences were limited to 1500 frames. The primary endpoint was measurement of flow rate for individual red cells within the vessel. We therefore used Imaris software (Bitplane Inc., South Windsor, CT), which allows tracking metrics of individual and populations of cells to be extracted. Following local background subtraction, fluorescent images of individual DsRed labeled erythrocytes were defined **(** **Fig. 1B**).

### Time-lapse, 3D Imaging of Vascular Reactivity

3D image stacks of *kdrl*-driven EGFP were collected using a Nikon Ti equipped with a Nikon sweptfield scan head, a Mad City Labs (Madison, WI) piezo driven Z-drive, and a 40X high NA (1.15 NA) long working distance water immersion objective. The sweptfield confocal pinhole used a 22 µm slit, and 25% laser power (equating to 8 mW at the end of the optical fiber for each color). 60 Z-positions were collected for each stack allowing a full stack to be collected in less than 1 s. Images were collected using NIS Elements and a Photometrics Evolve EMCCD camera (Photometrics, Tucson AZ) using 10–30 ms exposures and EM gain setting of 700.

**Figure 4:**
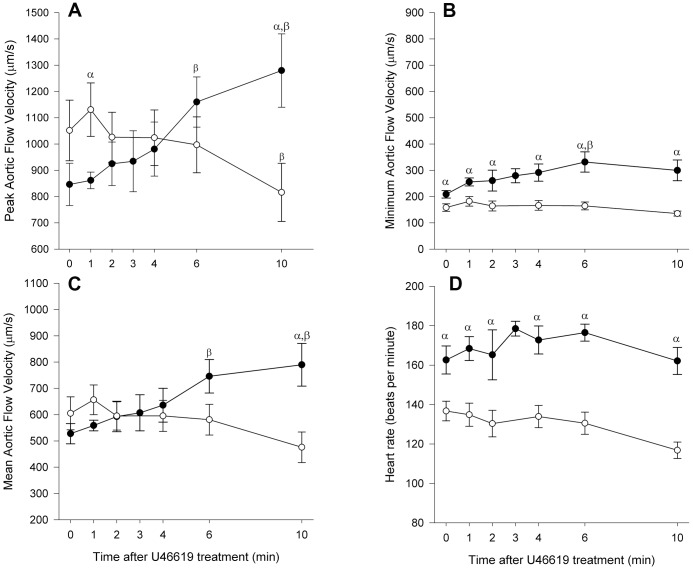
Comparison of aortic blood flow velocities in 3 dpf and 5 dpf zebrafish embryos. Plots show the mean data (± SE) for peak (**a**), minimum (**b**) and mean (**c**) aortic blood flow velocities and mean heart rate (**d**) in 3 dpf (open circles, n = 15) and 5 dpf (closed circles, n = 10) zebrafish embryos; and the changes in these parameters in response to the thromboxane A_2_ (TXA_2_) analogue, U-46619. α denotes statistical differences (ANOVA, *P*<0.05) between age groups; β denotes statistical differences from baseline (time 0).

### Time-lapse Imaging of Endothelial Barrier Function

By convention, vascular injections into either the cardinal vein or heart of embryonic zebrafish have been performed by tricane anesthesia and embedment in agarose. However, it was essential to be able to image at high resolution using multiple imaging modalities. Thus the embryos need to be mounted directly on the glass substrate at the base of a petri dish (35 mm, 14 mm microwell, MatTek Corporation) for use with high numeric aperture, short working distance lenses. As we wish to look at effects of vascular agonists, it was deemed important not to render the embryos hypoxic by embedment in agarose. We therefore developed methods for injection using immobilization paddles (**Fig. S1A**) designed in our laboratory and fabricated by Fotofab (Chicago, IL) using our original computer-aided designs (CAD, **Fig. S1B**). Two micromanipulators (Sutter 225, Sutter Instrument Company, Novato, CA) were used, one to hold the paddle, the other to hold the microinjection needle (**Fig. S1C**). Needles were pulled from borosilicate glass (B100-75-10, Sutter Instrument Company), and tips broken to an angle with forceps. The outside diameter of the tip measured 30–70 microns. Tips were loaded with a mix of 0.5 mg/ml rhodamine B isothiocyanate-dextran (70 kD) and 0.4%, 0.1 µm carboxylate-modified microspheres (Orange 540/560 FluoSpheres, Invitrogen) mixed with U-46619 (Cayman Chemical Company, final concentration in the needle was 29 µM) or 0.01 units/ml thrombin (Sigma-Aldrich). Needles were placed within the duct of Cuvier (common cardinal vein) of *chrna1^b107/+^;Tg(kdrl:GFP)^la116^* transgenics and small volumes (up to 25 ηL) of the bead/dextran mix were delivered. The rhodamine-labeled dextran was used to confirm that contents were flowing properly out of the pipette tip and into the lumen of the vessel. The concentration of beads in the injecting needle was kept to <0.5% as increasing the concentration beyond this led to vascular occlusion. At this concentration and volume there was no effect on vascular flow rate. Using a Nikon sweptfield confocal scan head, piezo driven Z-stage and a 10X high NA (0.5) objective, two color Z-stacks were collected (1–3 micron spacing, 50–100 Z-positions) with a Photometrics Evolve camera (20 Z-planes/second). A quad pass filter (Chroma Technology Corp., Bellows Falls, VT) was used for these studies in order to maximize speed. The illumination was scanned in sequential frame mode and there was no evidence of bleed through between the channels. Using these parameters, a complete 2-color stack was collected in 5 seconds. Z-stacks were collected every 2 minutes post-injection. The zebrafish were imaged for up to 2 hours with no motion, and good image registration.

**Figure 5:**
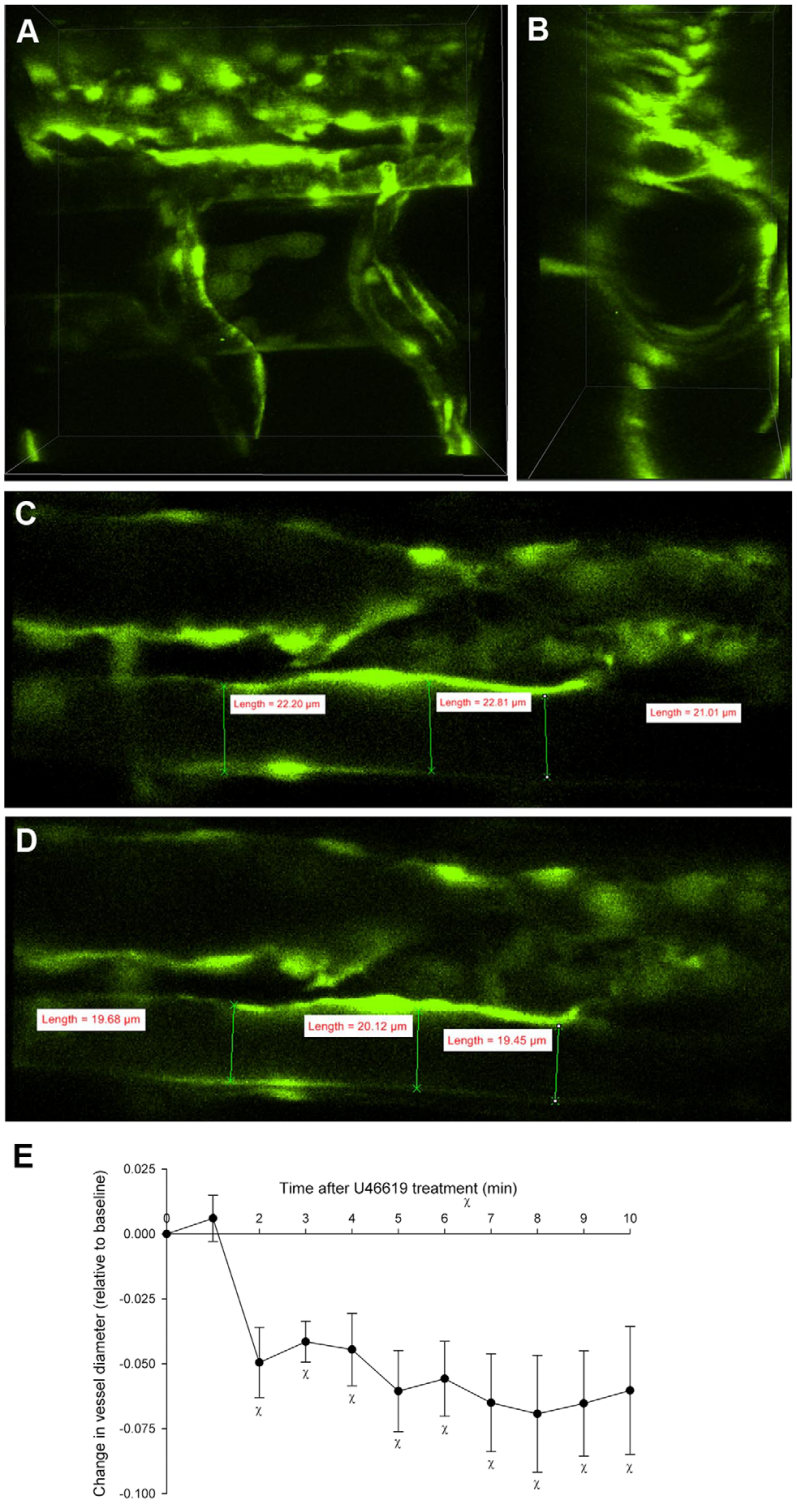
The thromboxane analogue, U-46619 decreased vessel diameter. 3D imaging of the dorsal aorta in a 5 dpf *Tg(kdrl:GFP)^la116^* zebrafish larva with dorsal downwards and anterior right (**A-D**). Changes in aortic flow velocity (Figure 5) in response to U-46619 in the 5 dpf embryos, were accompanied by decreases in vessel diameter (**E**, n = 5, *P*<0.05). χ denotes statistical differences from baseline (time 0).

**Figure 6:**
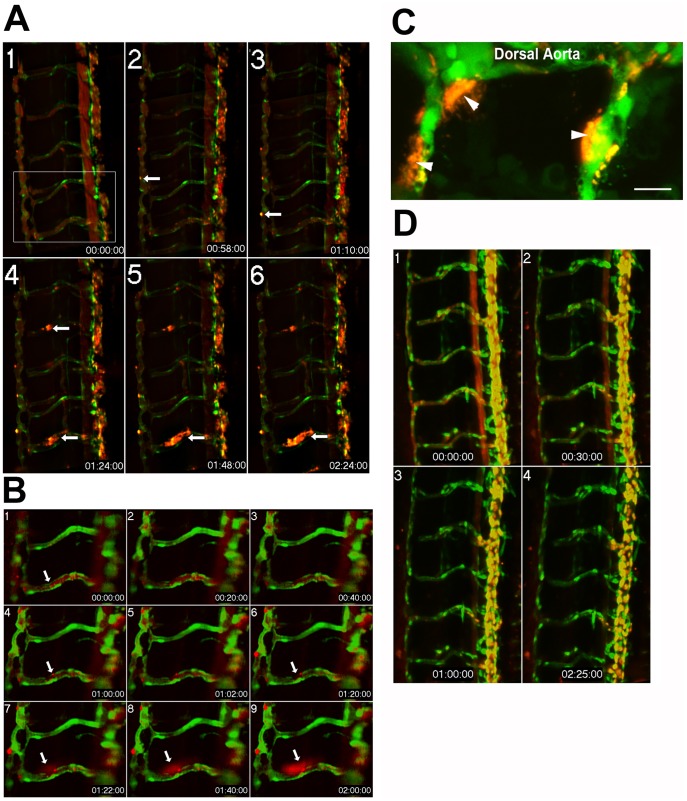
Time-lapse imaging of endothelial barrier dysfunction in response to the serine protease, thrombin. Extended depth of focus projection of the trunk vessels from a 5 dpf *Tg(kdrl:GFP)^la116^* zebrafish larva (**A**). The EGFP-labeled endothelium appears green while the 0.1 µm FluoSpheres are pseudocolored red. Data were collected at baseline and at 10 minute intervals following exposure to thrombin (0.01 units/µL) for a total collection period of 3 hours. Localized increases in permeability, as evidenced by accumulation of microspheres outside the vessel wall (white arrows) were apparent by 1 h (panel **2**) and increased over time (panels 3–6). The zoomed images (**B**) from boxed region of panel A1 show a clear break in the endothelial layer at 1 hour (panel 4). A total of 8 larvae were examined with equivalent results. These data were confirmed in a subset of larvae (n = 3) using high magnification (60X, 1.4 NA) point scanning confocal imaging and showed extensive accumulation of Fluospheres outside the vessel wall (**C**). In contrast, 5 dpf zebrafish larvae (n = 8) exposed to the thromboxane mimetic, U-46619 showed no evidence for disrupted barrier function (**D**).

### Statistical Analysis

Data were expressed as means ± SE and values compared using ANOVA with Student Newman–Keuls post hoc testing. An overall value of P≤0.05 was considered statistically significant.

## Results

### Real-time Visualization and Quantification of Blood Flow Velocity in Zebrafish Embryos

Scientific CMOS cameras have similar quantum efficiencies as conventional cooled CCD cameras in the spectral range being used here. As a result of the reduced read noise of the CMOS detector technology, it possible to read from these cameras much more rapidly than with other commercially available CCD devices. We were able to image a 1 mm section of the dorsal (**Fig. 1A**) aorta by reading entire columns of pixels at a time over 5–7 cardiac cycles at very high speed (486 fps) without the need for binning (**Fig. 1B** **)**. Due to the extremely rapid frame rate and short exposure times, the images of individual cells remained exact, without smearing as shown in the representative time sequence (**Fig. 2** **,** **Fig. 3A** **, movie S1**). Red blood cell flow velocity was quantified by first segmenting individual cells by size, shape and intensity (**Fig. 3B** **)**, followed by an autoregressive tracking algorithm which assumed a continued vector for travel (**Fig. 3C**). Autoregressive tracking is a commonly used approach in predictive fields ranging from motion analysis, as described here, to stock market predictions and analysis. The approach predicts the probability of future events in relation to past values. In this case the track that a particle (red blood cells) has followed predicts the next locale for that particle. Only cells that stayed in focus, and were tracked for at least 250 microns, were included in the analysis (total distance of collection was 0.6 mm to 1 mm). Red blood cells which did not move, or which moved into the segmental vessels were excluded from the final analysis. We were thereby able to generate instantaneous flow velocity profiles for individual erythrocytes over a 2–4 second period of measurement (example from 3 dpf and 5 dpf embryos shown in **Fig. 3D and 3E**, respectively). The mean velocity of all measured cells (25–30 cells per time point) was calculated at baseline, and at each time point following addition of the agonist. We obtained average peak and minimum aortic flow velocities of 1052±115 and 158±14 µm/s, respectively in 3 dpf (n  = 15) and 846±80 and 209±14 µm/s in 5 dpf (n  = 10) unanesthetized larvae (**Fig. 4A,B**). The measurements of blood flow reported in the literature vary depending on the speed and sensitivity of the measurement system; the requirement for aesthesia, embedment or cooling of the organism; and the age of the embryo. Measurements made using particle tracking methods relying on calculations of displacement between static consecutive frames of data, generally reflect mean aortic flow velocities. Our mean flow velocities of 605±63 and 527±39 µm/s (**Fig. 4C**) for the 3 dpf and 5 dpf embryos, respectively, are consistent with reported values [24], [25]. In agreement with other groups [24], [26], [27], we found that resting heart rate (estimated by extrapolation from the number of beats in the 10 s measurement interval) was higher in the older embryos (**Fig. 4D**) averaging 137±5 bpm and 162±7 bpm (*P*<0.05), for 3 dpf and 5 dpf embryos, respectively.

### Dynamic Regulation of Blood Flow by Thromboxane A_2_ in 3 and 5 dpf Zebrafish Larvae

In order to determine the quantitative capabilities of the imaging system to measure increases in blood flow velocity, we tested the response of 3 and 5 dpf zebrafish embryos to the stable thromboxane A_2_ (TXA_2_) analogue, U-46619. TXA_2_ is the main arachidonic acid metabolite generated by the sequential action of cyclooxygenase (COX) and thromboxane synthase (TXS); and has been shown to elicit diverse physiological effects in the vasculature system including smooth-muscle contraction and proliferation, endothelial dysfunction, and platelet activation and aggregation [28]. Zebrafish express both COX-1 and COX-2, phospholipase A2, and thromboxane A synthase (TXA synthase) [29]; and recent data support a role for the prostanoid synthesis pathway and COX2-TXA signaling in the regulation of mesencephalic blood flow by toxic environmental contaminants in 2 dpf zebrafish embryos [11]. We found that addition of U-46619 (1 µM) induced significant increases in peak aortic and mean blood flow velocity in 5 dpf embryos (n  = 10, *P*<0.05, **Fig. 3E** **,** **Fig. 4A and 4C**) with no change in minimum aortic flow velocity (**Fig. 4B**). In contrast, 3 dpf embryos showed a great deal of variability in their response to U-46619 with no overall change in aortic flow velocity or heart rate (n = 15, **Fig. 3D** **,** **Fig. 4**).

### Time-lapse, 3D Imaging of Vascular Reactivity in Response to Thromboxane A_2_

The degree of constriction or relaxation of a vessel (i.e. vessel diameter) is a key determinant of vascular resistance and as such, regulates regional blood flow. According to Poiseuille’s equation, flow is related to radius to the fourth power and thus very small decreases in vessel radius can dramatically reduce blood flow. Accurate real-time measurement of dynamic changes in vascular resistance, therefore, requires the ability to generate high speed, 3D reconstructions of the vascular network over time with sufficient resolution to detect small changes in diameter. Measurements derived from single plane images of the vessel wall are subject to errors caused by even very minute focal drifts in the Z-axis. The point scanning confocal and multiphoton microscopes typically used for optical sectioning are less suitable than multi-pinhole scan heads for live cell/tissue imaging because of the relatively slow scan speed along with the associated phototoxicity of these devices when compared with multi-pinhole systems. Even when used in resonant mode this frame rate is limited to 28 fps at high resolution. Furthermore, the currently available photomultiplier tube (PMT) detectors used in point scanning systems, have much lower quantum efficiencies (30%) compared with modern back-thinned electron multiplying CCD cameras (∼90%). We investigated whether the effects of U-46619 on blood flow velocity could be mediated by changes in vascular resistance by examining its effects on the diameter of the dorsal aorta in 5 dpf zebrafish embryos using high speed, slit-scanning confocal imaging. We were able to image the entire depth of 5 dpf larvae (**Fig. 5A, B**) using a 40X (1.15 NA) water immersion objective. In order to assess reactivity, the midplane of the vessel was selected (by maximum diameter measurement) and fiduciary markers were delineated at the image from time 0. Multiple (3–5) measurements were made at these fiduciary points across the vessel (**Fig. 5C, D**). The same measurements were made at each of the subsequent time points and the diameters were tabulated for each fish. The addition of U-46619 (1 µM) induced a mean decrease in diameter of 6.5±1.0% (n  = 5, P<0.05, **Fig. 5E**).

### Assessment of Endothelial Barrier Function in 5 dpf Zebrafish Larvae

In order to assess endothelial barrier function in the zebrafish larvae, we used fluorescence based time-lapse imaging techniques to track extravasion of fluorescently labeled tracer particles microinjected into the vascular space. We first tested our ability to measure disruption of barrier function using the coagulation serine protease, thrombin (29 µM). We observed leakage of fluorescent microspheres from the vascular space after exposure to thrombin in all 8 of the 5 dpf larvae studied (**Fig. 6A**). These changes in barrier function were accompanied by disruptions in endothelial continuity (**Fig. 6B**). We used high magnification (60X, 1.4NA) point scanning confocal imaging (Olympus Fluoview 1000) to confirm these results and showed that there was extensive accumulation of fluospheres outside the endothelial barrier (**Fig. 6C**). While thromboxane induced changes in contractility (**Fig. 5**), we did not observe any evidence for increased permeability in the U-46619 (1 µM) treated larvae we examined (n = 8, a representative image appears in **Fig. 6D**). In mammalian models, the effects of prostanoids on endothelial barrier function appear to be tissue-specific, with TXA_2_ receptor agonists inducing increases in vascular permeability in the aorta, lung, heart, kidney, spleen, and testis [30]. To our knowledge, the effects of U-46619 on endothelial permeability have not been previously studied in zebrafish.

## Discussion

We used high speed, multidimensional optical microscopy to dynamically assess metrics of vascular function (blood flow and vascular reactivity) in real time in unanaesthetized zebrafish embryos. Until recently, the high speed detectors required to make these measurements (video-rate and CCDs devices) were only fast enough to measure either slower peripheral blood flow, or flow after treatment to slow heartrate (i.e. anesthesia, cooling). Erythrocyte tracking methods such as digital motion analysis [14] and digital particle image velocimetry (DPIV) [12], [15], [16] have been used extensively to obtain gross measurements of blood flow and approximations of vessel diameters in developing zebrafish. In fact, an early example of applying DPIV to study early cardiac function in the developing heart tube of zebrafish embryos yielded data that contradicted the accepted model of peristalsis as the main pumping mechanism in the embryonic vertebrate heart [31]. However, as with traditional erythrocyte tracking methodologies, the utility of DPIV is limited by the temporal resolution of the camera and the ability of the system to resolve the particles within the flow field [17]. In contrast, the CMOS detector employed in the present studies enabled us to image a very large (1 mm) segment of the region of interest (in this case, the dorsal aorta of the zebrafish embryo) over many cardiac cycles, with sufficient speed and sensitivity that the trajectories of individual red blood cells could be resolved in real time. We feel that the system used here provides significant advantages over existing techniques, certainly from the point of view of the camera technology employed. But also, the use of a relatively high numeric aperture (NA) low magnification objective not only allows large areas to be imaged, it does so with a high photon recovery, and with a relatively large depth of field. Certainly higher NA objectives will provide a higher resolution, and perhaps brighter image, but the decreased field of view, and reduced axial focal volume makes these objectives much less attractive for this type of imaging, as erythrocytes moving axially within the vessel will move out of the focal plane. This approach allows a much higher event count at any time point. Using these methods, we were able to obtain flow velocity profiles over multiple cardiac cycles in 3 and 5 dpf zebrafish embryos, and assess dynamic changes in response to a TXA_2_ agonist (U-46619) which has well-characterized effects on vascular tone in other vertebrate models [30]. We propose that the described methods represent a simple, inexpensive, and reproducible approach to evaluate vascular performance in animal models of human disease and may be extended to an array of microscopic measurements that are currently limited by sensitivity and time resolution.

Zebrafish form fully lumenized vessels with intact barrier function in the dorsal aorta, cardinal vein and intersegmental vessels, as early as 54 hours post fertilization [32]. Endothelial permeability has been previously assessed using both wide-field and confocal methods to compare extravasation of fluorescent dextrans or microspheres from the vascular space at fixed time points, between treatment groups [32], [33], [34]. Here we use high resolution, time-lapse imaging to show real-time changes in permeability in response to a vasoactive agonist (thrombin), and further demonstrate that it is possible using high NA optics to identify regional changes in endothelial continuity. In future studies, these measurements can be combined with measurements of calcium flux, and pharmacological or genetic inhibition of key signaling pathways to provide mechanistic information about the pathways regulating endothelial dysfunction in models of human disease.

Beyond the technical advances described above, our observation that 5 dpf embryos regulate aortic blood flow in response to a vasoconstricting agent (U46619), at a point in development when there is only limited expression of vascular smooth muscle [35], suggests that endothelial constriction contributes to the regulation of vessel tone. While the heart and circulation of the zebrafish embryo are functional from 1 dpf, expression of embryonic smooth muscle cell markers is relatively weak and discontinuous throughout the length of the dorsal aorta even at 5 dpf [35], as we confirmed in our model using transmission electron microscopy (TEM, **Figure S2**). Vascular endothelium contains all the molecular machinery required to generate contractile force via the actomyosin motor, and has been shown to play a role in capillary constriction in a number of vascular beds in other vertebrate models [36], [37] ^,^ [38]. Furthermore, endothelial cells in early stage zebrafish embryos have been shown to produce vasoconstrictive agents including prostacyclin [11]; and nitric oxide (NO) has been shown to modulate vascular tone early in development when both nervous control and smooth muscle cells are limited or lacking [39], [40].

The actions of TXA2 are mediated by a G-protein-coupled receptor (TxA2 receptor) triggering activation of phospholipase Cβ via Gq/11, and ultimately inducing increases in intracellular Ca2+ and cell contraction [30]. While untested in this study, the observed age related differences in responsiveness to U-46619 could reflect developmental changes in the expression patterns of TXA receptors, or other proteins involved in the prostanoid/thromboxane signaling pathway. For example, the TXA synthase transcript does not become apparent until 3 dpf [41]. Relative changes in protein expression between the hatching period (3 dpf) and larval stage (5 dpf) have been described for a number of canonical signaling proteins, including those related to calcium handling/signaling [42].

It is well recognized that the endothelium modulates vascular tone via production of vasoactive mediators including endothelin and NO, while also acting as a semi-permeable barrier between the vascular lumen and surrounding tissue. Disruption of the endothelial barrier has been shown to be mediated via Ca^2+^/calmodulin-dependent assembly of actin stress fibers, cell contraction, disruption of cell–cell contacts, and intercellular gap formation [43]. The cellular effects of thrombin are mediated by protease-activated receptors (PARs), a subfamily of related G-coupled protein receptors (GCPRs) that are activated by cleavage of part of their extracellular domain. To date, four zebrafish *par* genes have been cloned and identified as homologs of mammalian *PARs1–3* [*44*]. While evidence for direct acute action of thrombin to increase permeability in intact vascular beds is limited [45], it has been previously shown that human thrombin can stimulate zebrafish Par1 to induce a Ca^2+^ response in *Xenopus* oocytes [46]. In these studies we used high speed slit scanning confocal microscopy to confirm that thrombin can induce time- dependent changes in endothelial barrier function *in situ* in 5 dpf zebrafish larvae.

Our data suggest that the scientific opportunities presented using an integrated suite of high speed imaging techniques are extensive, and are penetrant in attempting to answer temporally important questions in vascular physiology. A limitation of the approach used here is that it will work best on embryonic trunk vessels, because of the restriction in the depth of penetration of wide field, non-multiphoton microscopy. While the working distance of the 10X objective used in these studies would work on older embryos, the *chrna1* deficient transgenic model we have employed (which is paralyzed) will not live once nutrients from the yolk are exhausted. However, if measurements are made on anaesthetized fish, then the age of imaging can be extended out further, such that flow velocities in animals with varying degrees of vascular smooth muscle coverage can be compared.

## Supporting Information

Figure S1 **Immobilization paddles (A) were designed in our laboratory and fabricated by Fotofab (Chicago, IL) using our original computer-aided designs (CAD. B).** Two micromanipulators (Sutter 225, Sutter Instrument Company, Novato, CA) were used (**C**), one to hold the paddle (right), the other to hold the microinjection needle (left).
(TIF)

Figure S2 **Transmission electron microscopy shows that the trunk region of the dorsal aorta of the zebrafish embryo was devoid of smooth muscle cells.** This image, taken from the midpoint of the region of the aorta used for time based imaging, demonstrates that no smooth muscle cells are present around the vessel at 5 dpf. Skeletal muscle cells are clearly visible, as is the pronephros (PN). Erythrocytes (RBC) are abundant within the vessel, and endothelial cells are clearly evident. No smooth muscle cells can be detected. (Scale Bar  = 20 microns).
(TIF)

Movie S1 **The movie is divided into two repeating sections, each section shows the blood flow along the aorta over a 1.5 second period (the time lapse sequence has been slowed down 10 fold).** The first section simply shows the erythrocytes moving along the vessel. The pulsatile nature of the flow is readily apparent. In the second sequence, the red cells have been segmented out and are overlaid with round spots (Imaris, Bitplane Inc.) which are shown to faithfully track the erythrocyte passage along the vessel. It is these segmented spots that were used for the quantitative analysis of flow.
(MOV)
