# Supplementary figures and images for: High Resolution Imaging of Vascular Function in Zebrafish
Source: PLoS One. 2012 Aug 30;7(8):e44018. doi: 10.1371/journal.pone.0044018 (PMC3431338; doi:10.1371/journal.pone.0044018)

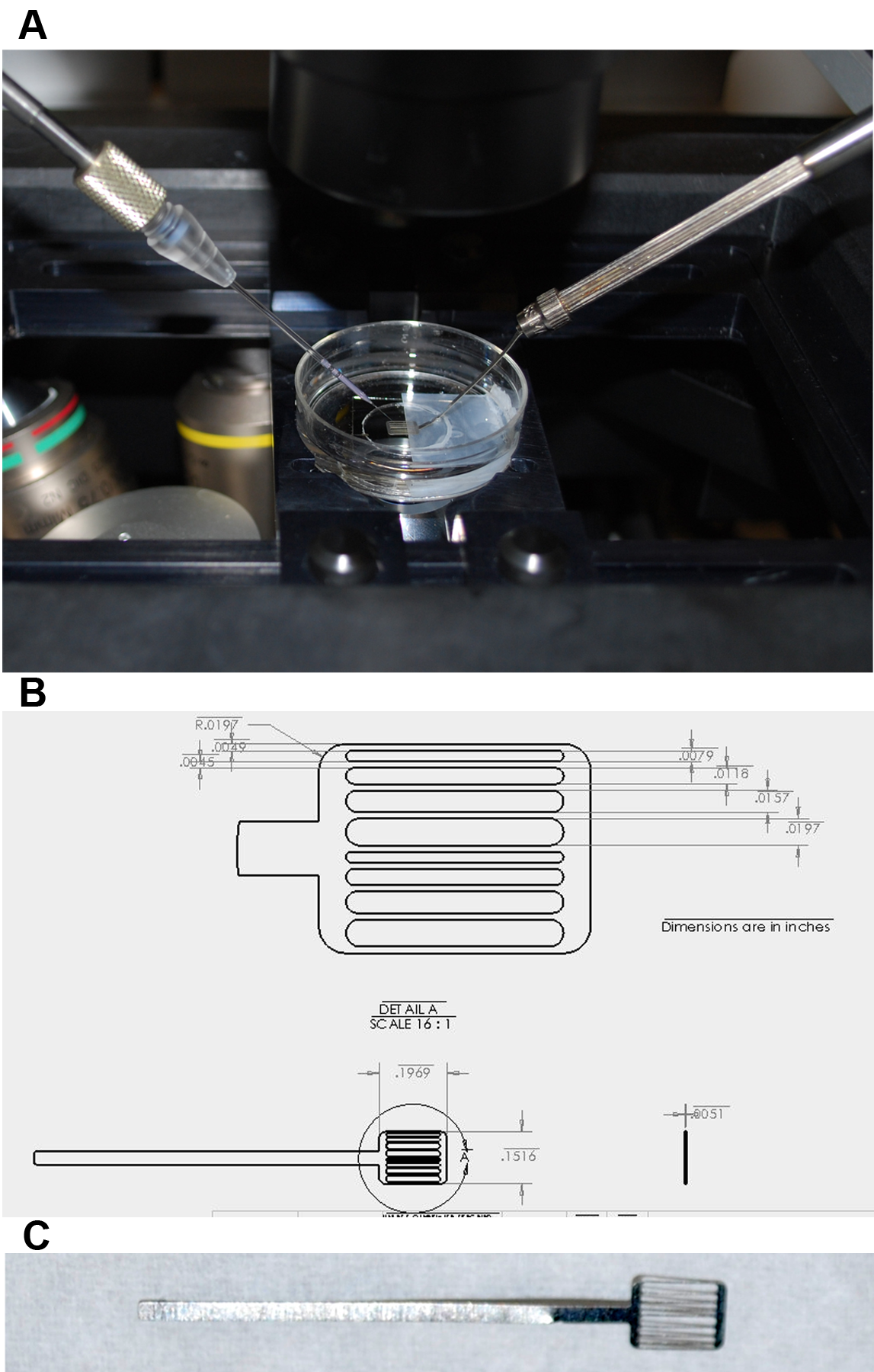

Supplement: Figure S1 — Immobilization paddles (A) were designed in our laboratory and fabricated by Fotofab (Chicago, IL) using our original computer-aided designs (CAD. B). Two micromanipulators (Sutter 225, Sutter Instrument Company, Novato, CA) were used (C), one to hold the paddle (right), the other to hold the microinjection needle (left). (TIF) [file pone.0044018.s001.tif]

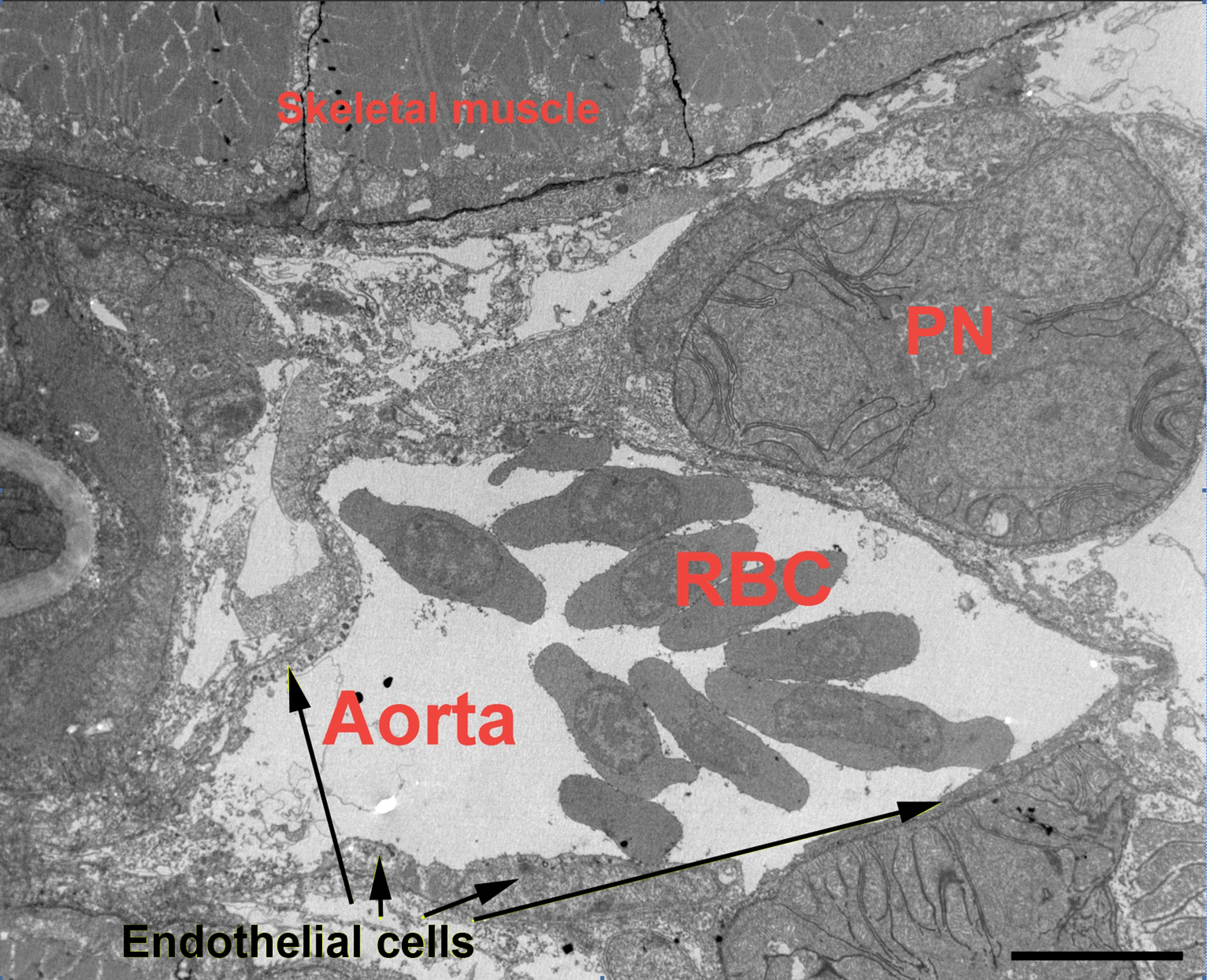

Supplement: Figure S2 — Transmission electron microscopy shows that the trunk region of the dorsal aorta of the zebrafish embryo was devoid of smooth muscle cells. This image, taken from the midpoint of the region of the aorta used for time based imaging, demonstrates that no smooth muscle cells are present around the vessel at 5 dpf. Skeletal muscle cells are clearly visible, as is the pronephros (PN). Erythrocytes (RBC) are abundant within the vessel, and endothelial cells are clearly evident. No smooth muscle cells can be detected. (Scale Bar = 20 microns). (TIF) [file pone.0044018.s002.tif]
